# Association of Serum Bilirubin Level with Metabolic Syndrome and Non-Alcoholic Fatty Liver Disease: A Cross-Sectional Study of 1672 Obese Children

**DOI:** 10.3390/jcm10132812

**Published:** 2021-06-25

**Authors:** Cristina Bellarosa, Giorgio Bedogni, Annalisa Bianco, Sabrina Cicolini, Diana Caroli, Claudio Tiribelli, Alessandro Sartorio

**Affiliations:** 1 Italian Liver Foundation, 34149 Trieste, Italy; giorgiobedogni@gmail.com (G.B.); annalisa.bianco@fegato.it (A.B.); ctliver@fegato.it (C.T.); 2 Life Science Department, University of Trieste, 34127 Trieste, Italy; 3 Istituto Auxologico Italiano, IRCCS, Experimental Laboratory for Auxo-Endocrinological Research, 28824 Verbania, Italy; s.cicolini@auxologico.it (S.C.); d.caroli@auxologico.it (D.C.); sartorio@auxologico.it (A.S.); 4 Istituto Auxologico Italiano, IRCCS, Division of Auxology and Metabolic Diseases, 28824 Verbania, Italy

**Keywords:** cross-sectional study, bilirubin, obesity, children, adolescents, metabolic syndrome, fatty liver, non-alcoholic fatty liver disease

## Abstract

As in adults, obesity also plays a central role in the development of metabolic syndrome (MS) in children. Non-alcoholic fatty liver disease (NAFLD) is considered a manifestation of MS. Not only MS but also NAFLD seem to be inversely associated with serum bilirubin concentrations, an important endogenous tissue protector when only mild elevated. The aim of the study was to evaluate the association between serum bilirubin levels and the prevalence of MS and NAFLD in Italian obese children and adolescents. A retrospective cross-sectional study was performed in 1672 patients aged from 5 to 18 years. Clinical and laboratory parameters were assessed. NAFLD was measured by liver ultrasonography. The study was approved by the Ethical Committee of the Istituto Auxologico Italiano (research project code 1C021_2020, acronym BILOB). MS was present in 24% and fatty liver (FL) in 38% of this population. Bilirubin was not associated with FL and MS as a whole, but it was inversely associated only with selected components of MS, i.e., large WC, high blood pressure and high triglycerides. Our data suggest that bilirubin is not protective against MS and NAFLD in the presence of severe obesity.

## 1. Introduction

The prevalence of overweight and obesity in children and adolescents has substantially increased in recent years [1]. As in adults, obesity also plays a central role in the development of metabolic syndrome (MS) [2,3] in children and adolescents. Metabolic syndrome is an important clustering of metabolic abnormalities and anthropometric characteristics entailing an increased risk for mortality from cardiovascular and all causes in adults [4,5,6], as well as an increase in type-2 diabetes and early cardiovascular disease in adolescence [7]. Using the IDF definition [8], the prevalence of MS among obese adolescents is comprised between 17 and 31% [9,10,11,12]. Non-alcoholic fatty liver disease (NAFLD) is presently considered a manifestation of MS [13,14]. There is increasing evidence that obesity, hyperglycemia and insulin resistance are risk factors for NAFLD in children [15,16,17]. Pooling data from studies performed mainly in tertiary care centers, the mean prevalence of NAFLD in children from general population studies was between 7.6% and 34.2% in studies based on child obesity clinics [18].

Bilirubin belongs to the tetrapyrrolic compounds superfamily and it is the end product of heme catabolism. Senescent erythrocytes are the principal sources of the heme group, together with the turnover of myoglobin, cytochromes, and other hemoproteins such as microsomal cytochrome CYP-450 [19]. Bilirubin is a water-insoluble compound that circulates bound to albumin and requires glucuronidation to be excreted by the microsomal uridine diphosphate glucuronosyltransferase (UGT) 1A1. Gilbert syndrome (GS) (also known as benign hyperbilirubinemia, OMIM No. #143500) is associated with mild unconjugated hyperbilirubinemia (>17 μM) due to decreased UGT1A1 activity. The prevalence of GS in the population is about 8%, and this syndrome is the most frequent cause of hereditary jaundice [20]. It is worth noting that interindividual variability in glucuronosylation of bilirubin by UGT1A1 is only partially explained by a genetic polymorphism because the penetrance of UGT1A1*28 allele homozygosity is only 50%, and epigenetic modifications seem to account for a substantial part of this variability [21,22].

For a long time, bilirubin has been considered a useless waste product of heme metabolism at best, or a toxic compound, especially for the central nervous system, at worst. Although its excessive accumulation is neurotoxic in neonates, more recent data indicate that bilirubin, when only mildly elevated, serves as an important endogenous tissue protector and a physiological modulator of oxidative stress and chronic inflammation in MS [23,24,25]. Total serum bilirubin has been reported to be inversely associated with central obesity, insulin resistance, dyslipidemia, and hypertension, together culminating in the manifestation of T2DM and CVD, in Korean and Chinese adults [26,27,28]. In a recent Austrian case–control study of subjects with GS, benign hyperbilirubinemia was negatively associated with BMI, hip circumference, and serum lipids [29]. Circulating total bilirubin has been reported to be inversely and independently associated with adverse cardio-metabolic outcomes in most [30,31,32], although not all [33,34], cross-sectional and cohort studies. A recent cohort study employing Mendelian randomization, however, casts doubt on the causal association of serum total bilirubin with cardio-metabolic outcomes [35]. Little is known about the association of bilirubin with MS in children and adolescents. In non-obese children, total serum bilirubin was inversely associated with body fatness [36] and with the prevalence of MS [37]. In obese children, body fat percentage was shown to be a major predictor of total serum bilirubin levels independently of the UGT1A1*28 polymorphism [38].

Not only MS but also non-alcoholic fatty liver disease (NAFLD) [39] and non-alcoholic steatohepatitis (NASH) [40] seem to be inversely associated with serum bilirubin concentrations. In a series of children with biopsy-proven NAFLD, Puri et al. showed that 67% had evidence of NASH. On multivariable analysis, higher bilirubin levels were inversely associated with the probability of a histological diagnosis of NASH [41]. Despite this evidence, the relationship between circulating total bilirubin and incident non-alcoholic fatty liver disease (NAFLD) is uncertain. Recently, two different studies were performed in China [42] and the Netherlands [43], aiming to assess the association of total bilirubin with the risk of new-onset NAFLD, and investigate the causal nature of their association using a Mendelian randomization approach. Multivariable analysis showed that elevated levels of total bilirubin were not causally associated with NAFLD.

The objective of the present study was to evaluate the association between serum bilirubin levels and the prevalence of MS and NAFLD in a large sample of obese Italian children and adolescents followed at a tertiary care center for pediatric obesity.

## 2. Materials and Methods

### 2.1. Study Design

A retrospective cross-sectional study was performed using an already available dataset of obese children followed at our tertiary care center for the treatment of pediatric obesity. The children were admitted to the clinic to undergo a short-term structured multidisciplinary weight-loss program. The inclusion criteria were: (1) Caucasian ethnic group; (2) age ≤ 18 years; (3) BMI ≥ 95th percentile for age and sex according to Italian growth charts [44]; (4) availability of liver ultrasonography (LUS). The exclusion criteria were: (1) genetic or syndromic obesity; (2) treatment with any drug; (3) alcohol intake (any quantity); (4) hepatitis B virus (HBV) or hepatitis C virus (HCV) infection. The study was approved by the Ethical Committee of the Istituto Auxologico Italiano (research project code 1C021_2020, acronym BILOB) and was conducted in accordance with the Declaration of Helsinki. The parents or the legal guardians of the subjects, or the subjects themselves when aged 18 years, gave written informed consent to participate in the study.

### 2.2. Clinical Assessment

Pubertal status was classified in 5 stages according to Tanner [45]. Weight and height were measured following standard procedures [46]. BMI was calculated as weight (kg)/height (m)^2^. Standard deviation scores (SDS) of weight, stature, and BMI were calculated using Italian growth charts [44]. Waist circumference was measured at the midpoint between the last rib and the iliac crest [47]. Alcohol consumption was determined by interview with the children/adolescents and/or their parents. No patients were treated with hepatotoxic drugs or exposed to iron overloads.

### 2.3. Laboratory Assessment

HBV surface antigen and antibodies against HCV were measured to exclude hepatitis B and C [48]. Alanine aminotransferase (ALT), aspartate aminotransferase (AST), gamma-glutamyl transferase (GGT), alkaline phosphatase, total bilirubin, total cholesterol, high-density lipoprotein (HDL) cholesterol, low-density lipoprotein (LDL) cholesterol, triglycerides, and c-reactive protein (CRP) were measured using a Cobas 6000 analyzer (Roche Diagnostics, SPA, Monza, Italy). Blood pressure was measured using a sphygmomanometer following international guidelines. Metabolic syndrome (MS) was diagnosed using the criteria of the International Diabetes Federation (IDF) [49]. The homeostasis model assessment of insulin resistance (HOMA-IR) was used as a surrogate measure of insulin resistance [50].

### 2.4. Liver Ultrasonography

LUS was performed using standardized criteria [51,52]. Normal liver was defined as the absence of liver steatosis or other liver abnormalities. Mild steatosis was defined as slightly increased liver echogenicity with normal vessels and absent posterior attenuation; moderate steatosis as moderately increased liver echogenicity with partial dimming of vessels and early posterior attenuation; and severe steatosis as diffusely increased liver echogenicity with absence of visible vessels and heavy posterior attenuation. NAFLD was operationally defined as any degree of liver steatosis in the absence of HBV and HCV infection and alcohol intake.

### 2.5. Statistical Analysis

Most continuous variables were not Gaussian-distributed and all were reported as median (50th percentile) and interquartile range (IQR, 25th and 75th percentiles). Discrete variables were reported as number and proportion. Linear regression models were used to quantify the association of continuous outcomes (age, BMI SDS, waist/height ratio, insulin, HOMA-IR and log_e_CRP) with bilirubin. Logistic regression models were used to quantify the association of binary outcomes (fatty liver, MS and its components, i.e., large waist circumference, high blood pressure, high triglycerides, high glucose, and low HDL-cholesterol) with bilirubin [8]. An ordinal generalized linear model (OGLM) was used to quantify the association between fatty liver degree (0 = none; 1 = light; 2 = moderate; 3 = severe) and bilirubin [30]. We used fractional polynomials to test whether the association between the continuous and binary outcomes and bilirubin was linear, and found it to be so in all cases [53]. Statistical analysis was performed using Stata 16.1 (Stata Corporation, College Station, TX, USA).

## 3. Results

### 3.1. Measurements of the Study Subjects

Table 1 gives the measurements of the 1672 study subjects. They were aged from 5 to 18 years and were mostly post-pubertal. FL was present in 38% of them and MS in 24% of them.

### 3.2. Multivariable Association between Bilirubin, Age, Pubertal Status and Sex

**Figure 1:**
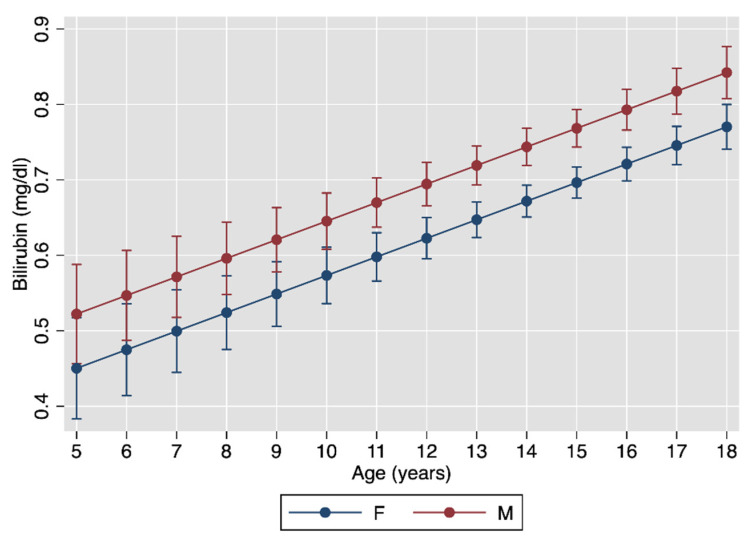
Association between bilirubin and age. See Table 2 for the underlying linear regression model.

**Figure 2:**
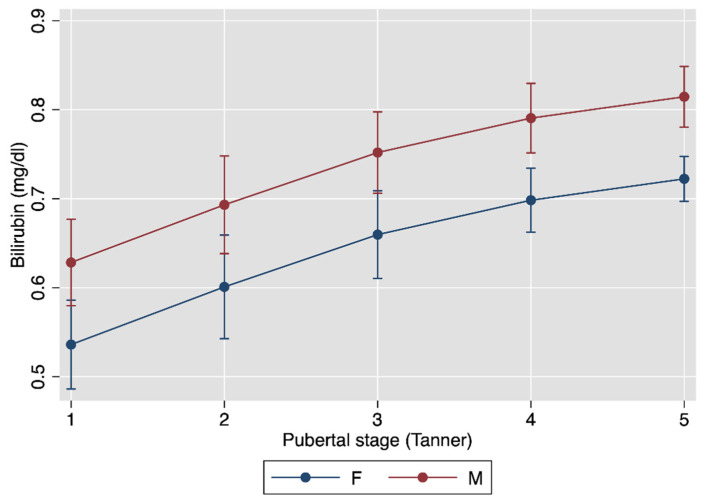
Association between bilirubin and pubertal status. See Table 2 for the underlying linear regression model.

Figure 1 plots the multivariable association between bilirubin, age and sex, and Figure 2 that between bilirubin, age and pubertal status, with the underlying regression models given in Table 2.

### 3.3. Univariable Associations between Bilirubin and Anthropometric and Laboratory Measurements

**Figure 3:**
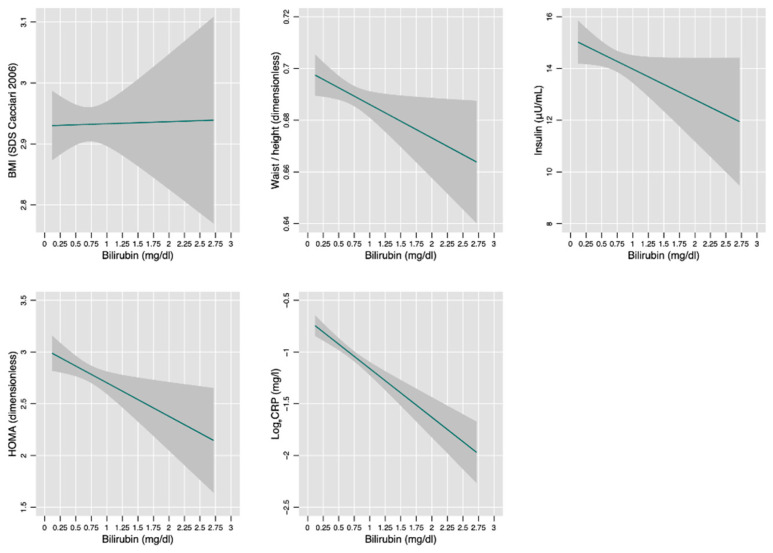
Association between bilirubin and anthropometric and laboratory measurements. See Table 3 for the underlying linear regression model.

Figure 3 plots the univariable associations between BMI (SDS), waist/height ratio, insulin, HOMA-IR, C-reactive protein and bilirubin. The corresponding linear regression models are given in Table 3.

Bilirubin was not associated with BMI and insulin, while it was inversely associated with weight:height ratio and CRP. However, the proportion of variance of weight:height ratio (0.2%) and log_e_CRP (2%) explained by bilirubin was very small and of no biological relevance.

### 3.4. Univariable Associations between Bilirubin, Fatty Liver, Metabolic Syndrome and Its Components

**Figure 4:**
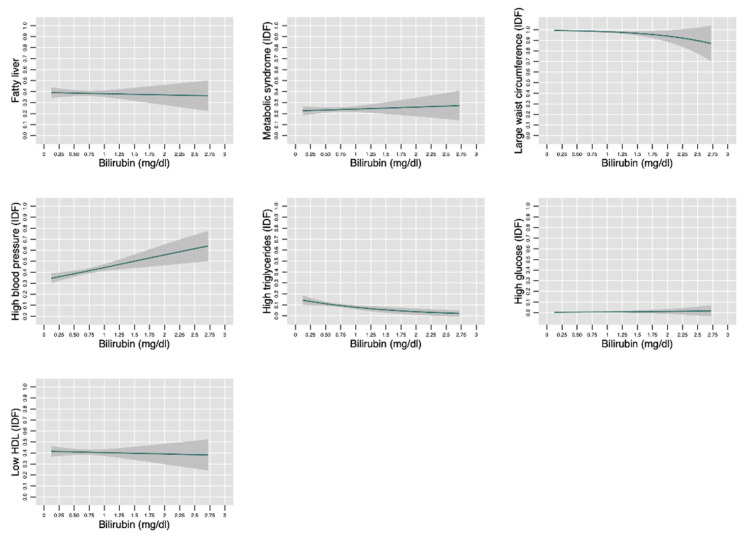
Association between serum total bilirubin, fatty liver, and the metabolic syndrome and its components. See Table 4 for the underlying logistic regression model.

Figure 4 plots the univariable associations between FL, MS and the single components of MS, i.e., large waist circumference, high blood pressure, high triglycerides, high glucose, and low HDL-cholesterol. The corresponding logistic regression models are given in Table 4.

Bilirubin was not associated with FL, MS, high glucose and low HDL, while it was inversely associated with large waist circumference, high blood pressure, and high triglycerides. However, as can be clearly seen from the probability plots given in Figure 4, these associations were weak, with the possible exception of that with high blood pressure, which had, nonetheless, very wide confidence intervals and was, thus, very imprecise at the higher values of bilirubin.

### 3.5. Univariable Association between Fatty Liver Degree and Bilirubin

**Figure 5:**
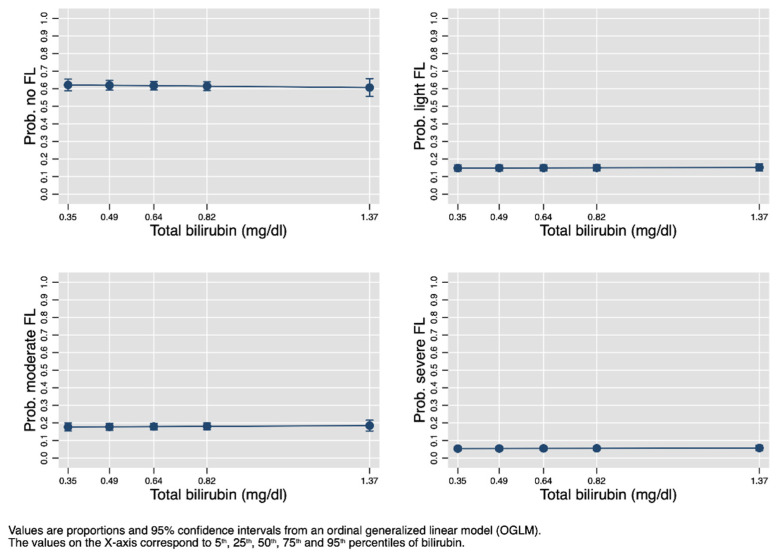
Association between serum total bilirubin and fatty liver degree.

Figure 5 plots the univariable association between FL degree and bilirubin as estimated by OGLM (model not shown). The figure clearly shows that the frequency of all degrees of fatty liver (none, light, moderate, and severe) is constant across a range of bilirubin values comprised between the 5th and the 95th internal percentiles.

## 4. Discussion

In the present study, performed on 1672 obese subjects aged 5 to 18 years, we tested whether bilirubin is associated with FL and MS and its components. Bilirubin was not associated with fatty liver categorized both as binary (yes vs. no) and ordinal (none vs. light vs. moderate vs. severe) (Table 4 and Figure 5). Bilirubin was also not associated with MS as a whole, but it was inversely associated only with selected components, i.e., large WC, high blood pressure, and high triglycerides (Table 4).

Bilirubin levels increased with increasing age and pubertal status and were higher in boys than in girls. These findings are in line with those obtained in the white subjects aged 5 to 30 years studied by the Bogalusa Heart Study [36]. Serum bilirubin levels were also found to be higher in US men than women [54]. As the gender disparity in serum bilirubin does not manifest before 10 years of age [36], it has been postulated that hormonal changes at puberty might change bilirubin metabolism [55]. This theory is supported by the fact that testosterone suppresses hepatic UGT activity in orchiectomized rats, while the combination of estradiol and progesterone enhances enzyme activity in gonadectomized female rodents [56].

Bilirubin levels were inversely associated with waist:height ratio (*p* < 0.05), HOMA-IR (*p* < 0.05), and CRP (*p* < 0.001) (Table 3). However, these associations were weak and of doubtful clinical relevance. An inverse association between serum bilirubin and HOMA-IR has been, nonetheless, previously reported in adults and children [57,58].

Chronic systemic inflammation plays a major role in the pathogenesis of MS-associated disease [59]. The serum levels of the inflammatory marker CRP are associated with all components of the MS and with insulin resistance, endothelial dysfunction, and impaired fibrinolysis [59]. It is likely that serum bilirubin protects against MS. In the obese subjects of the Belo study [38], total serum bilirubin was inversely correlated with CRP (r = −0.178, *p* = 0.001). The negative association that we detected between bilirubin and CRP, even with the normal range of the latter, is in line with these findings and the anti-inflammatory activity of bilirubin [60,61,62,63].

A mechanism linking bilirubin and plasma lipids has been proposed [64]. Reduced circulating total cholesterol [65], LDL-C [66], TAG [67], and elevated HDL/LDL ratio [68] have been reported in mild hyperbilirubinemic adult Gilbert syndrome subjects. An inverse association between Ox-LDL [69], triglycerides [36], and serum bilirubin was also shown in young obese patients. Similarly, we detected an inverse correlation between total serum bilirubin levels and high triglycerides (*p* < 0.001) (Table 4).

Little is known about the association between bilirubin and MS in children and adolescents. In a representative sample of the non-institutionalized civilian US population (NHANES), Lin et al. examined 4723 children and adolescents aged 12 to 17 years and identified 190 (4%) with MS. Serum total bilirubin was significantly higher in subjects without MS than in those with it. The authors speculated that in young subjects, when the metabolic dysregulation is in its early stages, bilirubin may exert its anti-oxidative effect within the tissues. Serum bilirubin levels, even within the normal range, might have graded associations with the prevalence of MS in children [37]. Similar results have also been reported in other studies conducted in Chinese [28,70], Korean [27,71], and Polish [57] adults.

In our study, total serum bilirubin was not associated with MS as a whole, but only with some of its components. The only available study of young obese children was performed in Portugal and showed an association between total bilirubin and percentage of body fat [38]. It is worth noting that the BMI mean ± SD was 30.7 ± 5.8 for females and 30.5 ± 6.4 for males. We think that the severe obesity of the cohort studied in the present work did not allow us to evidence the ability of serum bilirubin to modulate MS in young ages, compared to when the metabolic dysregulation is in its early stages.

In the present study, 38% of a large sample of obese children had NAFLD. Even if a prevalence of 38% is within the expected range [18], it is substantially lower than that observed in a series of Chinese children with a similar degree of obesity (77). This difference is of interest because it may be explained by genetic and/or environmental factors, similar to what has been hypothesized for adults [72,73]. In our cross-sectional study, bilirubin was not associated with fatty liver degree. The same association was investigated in a sample of Taiwanese obese children stratified by UGT1A1 genotype, where 12% had NAFLD [74]. Serum total bilirubin was lower in the children with NAFLD and an independent inverse association between variant UGT1A1*6 genotypes and NAFLD prevalence was reported. The main differences between the Taiwanese study and the present study is the number of participants (234 vs. 1672) and the severity of obesity, which was much higher for the present study.

The present study has some limitations. First, its results cannot be extended to the general pediatric population comprising of non-obese children. Second, as we have discussed in detail elsewhere [48,75], although LUS is the best technique for epidemiologic studies because of its availability and non-invasiveness, it works well for detecting fatty infiltrations of the liver ≥30%, thus possibly underestimating the prevalence of fatty liver.

## 5. Conclusions

In conclusion, in a population of severely obese children and adolescents, we did not detect any association between serum total bilirubin, FL and MS as a whole. Bilirubin was nonetheless inversely associated with HOMA-IR, CRP, and with three components of MS, i.e., large WC, high blood pressure, and high triglycerides. Although these associations were statistically significant, they cannot be considered biologically relevant on the basis of the underlying effect size. Our data seem to be in contrast with the available literature showing an inverse association between bilirubin and MS. Conversely, our data adds something new, focusing on the grade of obesity and suggesting that bilirubin is not protective against MS and NAFLD in the presence of severe obesity. 

## Figures and Tables

**Table 1:** Measurements of the study children. Abbreviations: BMI = body mass index; SDS = Standard deviation score); and interquartile range (IQR, 25th and 75th percentiles). Discrete variables are reported as the number and proportion of subjects with the characteristic of interest.

	Total
	N = 1672
Sex	
F	980 (58.6%)
M	692 (41.4%)
Age (years)	15 (13; 16)
Pubertal stage (Tanner)	
1	194 (11.6%)
2	144 (8.6%)
3	213 (12.7%)
4	373 (22.3%)
5	748 (44.7%)
Weight (kg)	96 (83; 112)
Weight (SDS)	3.01 (2.47; 3.57)
Height (m)	1.63 (1.56; 1.69)
Height (SDS)	0.33 (−0.30; 1.04)
BMI (kg/m^2^)	36 (32; 40)
BMI (SDS)	2.92 (2.50; 3.32)
Waist circumference (cm)	111 (101; 122)
Large waist circumference (IDF)	1646 (98.4%)
Waist/height (dimensionless)	0.68 (0.63; 0.74)
ALT (U/L)	23 (16; 35)
AST (U/L)	21 (17; 26)
GGT (U/L)	16 (12; 22)
Alkaline phosphatase (U/L)	164 (91; 263)
Total bilirubin (mg/dL)	0.64 (0.49; 0.82)
Glucose (mg/dL)	79 (74; 83)
Insulin (μU/mL)	13 (9; 18)
HOMA-IR (dimensionless)	2.4 (1.6; 3.5)
Cholesterol (mg/dL)	162 (142; 182)
HDL-cholesterol (mg/dL)	43 (37; 51)
Low HDL (IDF)	681 (40.7%)
LDL-cholesterol (mg/dL)	102 (85; 122)
Triglycerides (mg/dL)	87 (66; 114)
High triglycerides (IDF)	163 (9.7%)
CRP (mg/L)	0.4 (0.2; 0.7)
Systolic blood pressure (mm hg)	120 (120; 130)
Diastolic blood pressure (mm hg)	80 (70; 80)
High blood pressure (IDF)	687 (41.1%)
Fatty liver	642 (38.4%)
Fatty liver degree	
None	1030 (61.6%)
Light	250 (15.0%)
Moderate	300 (17.9%)
Severe	92 (5.5%)
Metabolic syndrome (IDF)	395 (23.6%)

**Table 2:** Multivariable linear regression models evaluating the association between serum total bilirubin and age and pubertal status.

	Total Bilirubin (mg/dL)	Total Bilirubin (mg/dL)
Age (years)	0.02 ***	
	[0.02,0.03]	
F	0.00	0.00
	[0.00,0.00]	[0.00,0.00]
M	0.07 ***	0.09 ***
	[0.04,0.10]	[0.06,0.13]
Tanner stage 1		0.00
		[0.00,0.00]
Tanner stage 2		0.06
		[−0.01,0.14]
Tanner stage 3		0.12 ***
		[0.06,0.19]
Tanner stage 4		0.16 ***
		[0.10,0.22]
Tanner stage 5		0.19 ***
		[0.13,0.24]
Intercept	0.33 ***	0.54 ***
	[0.23,0.43]	[0.49,0.59]
N	1672	1672
R-squared	0.038	0.037

Values are linear regression coefficients and 95% confidence intervals (in brackets). *** *p* < 0.001.

**Table 3:** Univariable linear regression models evaluating the association between serum total bilirubin and anthropometric and laboratory measurements. Abbreviations as in Table 1.

	BMI	Waist/Height	Insulin (μU/mL)	HOMA-IR (Dimensionless)	Log_e_CRP (U/mL)
Total bilirubin (mg/dL)	0.00	−0.01 *	−1.18	−0.32 *	−0.47 ***
	[−0.08,0.09]	[−0.02,−0.00]	[−2.40,0.03]	[−0.57,−0.07]	[−0.62,−0.32]
Constant	2.93 ***	0.70 ***	15.16 ***	3.03 ***	−0.69 ***
	[2.86,3.00]	[0.69,0.71]	[14.20,16.12]	[2.83,3.22]	[−0.80,−0.57]
Observations	1672	1672	1672	1672	1672
Adjusted R-squared	0.001	0.002	0.002	0.003	0.023

Values are logistic regression coefficients and 95% confidence intervals in brackets. * *p* < 0.05, *** *p* < 0.001.

**Table 4:** Univariable logistic regression models evaluating the association between serum total bilirubin, fatty liver, and the metabolic syndrome and its components.

	Fatty Liver	Metabolic Syndrome (IDF)	Large Waist Circumference (IDF)	High Blood Pressure (IDF)	High Triglycerides (IDF)	High Glucose (IDF)	Low HDL (IDF)
Total bilirubin (mg/dL)	−0.05	0.10	−1.17 **	0.46 **	−0.77 **	0.53	−0.05
	[−0.34,0.25]	[−0.24,0.43]	[−1.96,−0.38]	[0.17,0.76]	[−1.35,−0.18]	[−1.02,2.09]	[−0.35,0.24]
Intercept	−0.44 ***	−1.24 ***	5.08 ***	−0.69 ***	−1.70 ***	−5.51 ***	−0.34 **
	[−0.67,−0.21]	[−1.51,−0.98]	[4.27,5.89]	[−0.93,−0.46]	[−2.12,−1.28]	[−6.88,−4.14]	[−0.57,−0.11]
*N*	1672	1672	1672	1672	1672	1672	1672

Logistic regression coefficients and 95% confidence intervals in brackets. ** *p* < 0.01, *** *p* < 0.001.

## Data Availability

Data sharing not applicable.
